# Evaluating Risk Factors and the Burden of Silent Myocardial Ischemia Among Diabetic Patients

**DOI:** 10.7759/cureus.74341

**Published:** 2024-11-24

**Authors:** Muhammad Muneeb Arshad, Muhammad Adeel Hassan, Muhammmad Tahir, Muhammad Shahid Nawaz khan, Muhammad A Gultasib, Gohar Ali

**Affiliations:** 1 Internal Medicine, University Hospital Birmingham National Health Service (NHS) Foundation Trust, Birmingham, GBR; 2 Cardiology, Nishtar Medical College, Multan, PAK; 3 Acute Medicine, University Hospital Southampton National Health Service (NHS) Foundation Trust, Birmingham, GBR; 4 Medicine, Nishtar Hospital Multan, Multan, PAK; 5 Medicine, Tertiary Care Hospital Nishtar, Multan, PAK; 6 Acute Medicine, University Hospital Birmingham, Birmingham, GBR; 7 Acute Medicine, Ayub Teaching Hospital Abbottabad, Abbottabad, PAK

**Keywords:** cardiovascular risk, diabetes, microalbuminuria, predictors, prevalence, silent myocardial ischemia

## Abstract

Introduction: Silent myocardial ischemia (SMI) is a significant concern for diabetic patients, often remaining undetected until severe complications arise. Prolonged hyperglycemia, poor glycemic control, and lifestyle factors contribute to its risk, with older adults and those with long-standing diabetes particularly vulnerable.

Objective: To assess the prevalence and predictors of SMI in adults with diabetes, emphasizing long-term management and monitoring.

Methodology: A longitudinal observational study was conducted at Nishtar Medical University, Multan, from October 2020 to September 2022, involving 388 adults with diabetes for at least five years. Patients with significant cardiovascular diseases or recent medication-affecting biomarkers were excluded. Data collection included demographics, medical history, and clinical assessments such as HbA1c levels, 12-lead resting ECGs, and treadmill exercise stress tests, with myocardial perfusion imaging (MPI) for those unable to perform stress tests. Statistical analyses using IBM SPSS (version 22) incorporated univariate and multivariate logistic regression to identify predictors of SMI, adjusting for confounders such as age, sex, smoking, and blood pressure. Thresholds included microalbuminuria at 30-300 mg/24 hours and HbA1c ≥7%, with missing data addressed through multiple imputations.

Results: Among the participants, the prevalence of SMI was 48%, increasing to 58% in those with microalbuminuria. Significant predictors included the duration of diabetes (OR 1.28, 95% CI: 1.08-1.52, p=0.001), HbA1c levels (OR 1.75, 95% CI: 1.42-2.16, p<0.001), age (OR 1.05, 95% CI: 1.01-1.09, p=0.035), and smoking status (OR 1.42, 95% CI: 1.05-1.92, p=0.025). Smoking status was based on self-report. Notably, microalbuminuria showed a strong association with SMI (OR 2.89, 95% CI: 2.10-3.98, p<0.001). The distribution of participants was balanced in terms of age and gender, with a mean age of 58 years (SD 9.4), and 52% were male. No unexpected findings were observed, and the results aligned with the anticipated relationships between the variables.

Conclusion: This study highlights a concerning prevalence of SMI among diabetic patients, emphasizing the importance of monitoring diabetes duration and glycemic control, particularly in individuals with microalbuminuria. Regular follow-up care, including routine ECGs, stress tests, and biomarker assessments, is crucial for improving cardiovascular outcomes in this high-risk population. Limitations such as the observational design and potential self-report bias in smoking status may affect the generalizability of the findings. Future research should focus on large-scale, multicenter studies to validate these findings and explore interventions that could reduce the burden of SMI in diabetic patients.

## Introduction

Silent myocardial ischemia (SMI) presents a significant concern among diabetic patients [1], often going undetected until severe complications arise. Research indicates that the prevalence of SMI in individuals with diabetes in Pakistan can reach as high as 48%, with even higher rates observed in those with additional risk factors [2]. Previous studies have shown that diabetic patients are at an increased risk for cardiovascular events due to factors like prolonged hyperglycemia, which contributes to microvascular and macrovascular complications. Specifically, chronic hyperglycemia leads to endothelial dysfunction and impaired myocardial perfusion, both of which are key mechanisms underlying the development of SMI in diabetic patients. Moreover, hyperglycemia accelerates the progression of atherosclerosis, further increasing the risk of ischemia [3]. Khanal et al. [4] highlighted the correlation between poor glycemic control and the increased incidence of ischemic heart disease, emphasizing the need for ongoing monitoring and early intervention in diabetic populations. Demographic variables, such as age and sex, also interact with other risk factors to influence SMI risk. For instance, older adults, particularly those with long-standing diabetes, exhibit higher rates of cardiovascular complications, which are compounded by poor glycemic control and the presence of microalbuminuria, further reinforcing the necessity for age-specific risk assessments [5,6].

The association between glycemic control and SMI is particularly well-documented, with elevated HbA1c levels serving as a critical marker of long-term glucose management. Better glycemic control has been shown to reduce cardiovascular disease risk in diabetics, making it essential for mitigating SMI risk [7]. A study by Mazzone [8] emphasized the role of structured diabetes management programs, showcasing that intensive glucose-lowering strategies can decrease cardiovascular events. Smoking is another modifiable risk factor strongly associated with an increased likelihood of SMI. Smoking cessation programs could play a significant role in reducing SMI risk, particularly in diabetic populations in Pakistan, where smoking prevalence remains a public health concern [9]. In addition, microalbuminuria, which reflects systemic vascular damage and endothelial dysfunction, has a strong association with SMI. This emphasizes the importance of regularly monitoring kidney function as a predictor for cardiovascular risks in diabetic patients [10].

From a Pakistani perspective, research on SMI among diabetic patients is limited, presenting a significant gap in understanding the local prevalence and associated risk factors. The growing incidence of diabetes in Pakistan, compounded by limited access to healthcare and preventative services, calls for a focused investigation into cardiovascular risks in this population. Lifestyle factors, such as dietary habits (e.g., high carbohydrate intake and low fruit and vegetable consumption) and physical inactivity, prevalent in Pakistani culture, may further exacerbate the risk of SMI among diabetic patients [10]. Additionally, the lack of comprehensive data on microalbuminuria prevalence and its correlation with SMI highlights the urgent need for multicenter studies in Pakistan to address these gaps. Such research could inform targeted interventions and healthcare policies to mitigate the rising burden of cardiovascular diseases in the country’s diabetic population.

## Materials and methods

The study was approved by the Institutional Ethical Review Board (IERB) of Nishtar Medical University, Multan (Approval No. 15226/NMU & H), dated August 12, 2020. The study duration was two years, from October 2020 to September 2022. Ethical considerations were prioritized, and participants received comprehensive information regarding the study's aims, procedures, risks, and confidentiality assurances. Informed consent was obtained from all participants, ensuring compliance with ethical guidelines for data confidentiality and risk minimization. Patients diagnosed with SMI, defined as asymptomatic myocardial ischemia detected through objective methods such as ECG abnormalities, were referred to a cardiologist for appropriate management and follow-up care. The rationale for follow-up intervals (every 3-6 months) was based on the need for consistent monitoring and timely interventions, taking into account the individual needs and health status of the patients. The follow-up care included adjustments in therapeutic interventions based on clinical progress and was tailored to each participant’s specific needs, including lifestyle modifications such as diet and physical activity recommendations. Common therapeutic interventions included medications like statins, beta-blockers, and antidiabetic agents, which were administered or adjusted based on clinical necessity.

The study adopted a longitudinal observational design to assess the prevalence of SMI among diabetic patients and to identify associated predictors over time. This design allowed for repeated measurements and follow-ups to monitor changes in health status and predictors of SMI within the same individuals.

The study population consisted of adults aged 40 to 75 with a confirmed diagnosis of diabetes for at least five years. Exclusion criteria included a history of symptomatic coronary artery disease or myocardial ischemia and the presence of comorbidities such as advanced renal failure, defined by an estimated glomerular filtration rate (eGFR) of less than 30 mL/min/1.73m², or recent use of medications that could significantly alter cardiovascular biomarkers. The final sample size was calculated using the G*Power method to ensure sufficient statistical power while maintaining a significance level of 0.05. Based on a recent study from Pakistan reporting a prevalence of 36.7% for SMI among diabetic patients [11], a prevalence of 40% in the general population, and 50% in those with microalbuminuria, the required sample size was calculated to be approximately 385 participants.

For recruitment, stratified random sampling was employed to ensure representation across key demographic variables, including age, sex, and duration of diabetes. A total of 388 participants were recruited from diabetic outpatient clinics and community health centers over an 18-month period.

Data collection involved obtaining patient demographics, medical history, smoking status, physical activity, and family history of cardiovascular diseases. Medication use, particularly antidiabetic, antihypertensive, and lipid-lowering drugs, was recorded. Clinical assessments included physical examinations, blood pressure, BMI, waist circumference, and laboratory tests measuring HbA1c, lipid profiles (total cholesterol, LDL, HDL, triglycerides), and inflammatory markers such as C-reactive protein and fibrinogen.

To detect SMI in diabetic patients, a structured diagnostic approach was employed. All participants underwent a 12-lead resting ECG as the first step in detecting ischemic changes. ECG abnormalities, such as ST-segment depression or T-wave inversions, were used as primary indicators of ischemia. If the resting ECG results were abnormal, patients were further subjected to a treadmill exercise stress test to assess for exercise-induced ischemia. In cases where patients were unable to perform the treadmill test due to physical limitations, pharmacological stress testing using agents such as adenosine or dobutamine was utilized to induce stress and evaluate myocardial perfusion. This comprehensive approach ensured the accurate detection of both symptomatic and asymptomatic ischemia, with multiple objective testing methods to confirm the presence of silent myocardial ischemia. These diagnostic protocols were aligned with current guidelines for the detection of ischemia in high-risk populations, ensuring robust detection of SMI in this cohort.

Data were analyzed using IBM Corp. Released 2013. IBM SPSS Statistics for Windows, Version 22.0. Armonk, NY: IBM Corp. The prevalence of SMI was calculated as the proportion of patients diagnosed with ischemia based on ECG or stress test results. Confidence intervals were reported for prevalence estimates to reflect statistical uncertainty. Univariate analyses, including chi-square tests for categorical variables and t-tests for continuous variables, were conducted to identify predictors of SMI, such as diabetes duration, HbA1c levels, and BMI. For continuous variables, one-way ANOVA was used, with post-hoc comparisons performed using Tukey's test to identify differences between groups. Multivariate logistic regression was employed to assess the independent predictors of SMI, adjusting for confounders such as age, sex, smoking status, and blood pressure. Wald chi-square tests were used within the logistic regression framework to evaluate the significance of predictors, and goodness-of-fit was assessed using the Hosmer-Lemeshow test (p=0.78), confirming adequate model fit. P-values less than 0.05 were considered statistically significant. All analyses were performed in SPSS, and the results were interpreted to account for potential biases and confounders.

To reduce observer bias, blinding of those interpreting ECG results was employed. Selection bias was minimized by recruiting participants from both outpatient clinics and community health centers, ensuring a broad representation of the diabetic population. Objective diagnostic tools, such as ECG and pharmacological stress testing, were used to minimize subjective bias in the diagnosis of SMI.

## Results

Table 1 presents the demographic and clinical characteristics of the 388 diabetic participants, categorized into two groups: those with silent myocardial ischemia (SMI positive, N=186) and those without (SMI negative, N=202). The average age of participants was 60.4 years, with SMI-positive individuals being older on average (61.3 years) compared to their SMI-negative counterparts (59.6 years). This difference was statistically significant (p=0.045).

**Table 1:** Demographic and clinical characteristics of participants

Variable		Total (N=388)	SMI Positive (N=186)	SMI Negative (N=202)	p-value	Test
Age (years)		60.4 ± 8.2	61.3 ± 7.9	59.6 ± 8.4	0.045	ANOVA
Gender	Male (n; %)	202; 52%	108; 58%	94; 48%	0.222	Chi-square
	Female (n; %)	186; 48%	78; 42%	108; 52%		
Duration of Diabetes (years)		10.5 ± 4.3	11.2 ± 4.1	10.0 ± 4.5	0.022	ANOVA
HbA1c (%)		8.5 ± 1.3	9.1 ± 1.2	7.9 ± 1.1	<0.001	ANOVA
BMI (kg/m²)		28.6 ± 4.7	29.1 ± 4.5	28.2 ± 4.8	0.058	ANOVA
Smoking Status (current smoker)		93; 24%	56; 30%	37; 19%	0.034	Chi-square
Family History of CAD (%)		155; 40%	84; 45%	71; 36%	0.112	Chi-square
Medication Use (Yes) (%)		252; 65%	130; 70%	122; 60%	0.045	Chi-square
Physical Activity (active) (%)		155; 40%	65; 35%	90; 45%	0.075	Chi-square

The duration of diabetes was significantly longer in the SMI-positive group (11.2 years) than in the SMI-negative group (10.0 years), with a p-value of 0.022, indicating that prolonged diabetes is associated with an increased risk of SMI. Hemoglobin A1c (HbA1c) levels, an indicator of long-term glucose control, were notably higher in the SMI-positive group (9.1%) compared to the SMI-negative group (7.9%), with a highly significant p-value of <0.001.

Body mass index (BMI) was slightly higher in the SMI-positive group (29.1 kg/m²) compared to the SMI-negative group (28.2 kg/m²). However, this difference was not statistically significant (p=0.058). While the result was not significant, the potential clinical relevance of higher BMI in predicting SMI warrants further exploration, particularly in high-risk diabetic populations.

Smoking status was more prevalent among SMI-positive participants (56/186, 30%) compared to SMI-negative participants (37/202, 19%), with a p-value of 0.034. This suggests that smoking may be a contributing factor for SMI. Similarly, physical activity levels were lower in the SMI-positive group (65/186, 35%) compared to the SMI-negative group (90/202, 45%), with a p-value of 0.075. Although not statistically significant, this trend indicates that reduced physical activity could potentially be associated with SMI, emphasizing the need for further research in this area.

A family history of coronary artery disease (CAD) was reported in 84/186 (45%) of SMI-positive patients compared to 71/202 (36%) of SMI-negative patients, though this difference was not statistically significant (p=0.112). Medication use was prevalent in 130/186 (70%) of SMI-positive individuals compared to 122/202 (60%) in the SMI-negative group, with a significant p-value of 0.045, highlighting a potential link between medication adherence and SMI risk.

The gender distribution in the study showed that 202/388 (52%) participants were male, and 186/388 (48%) were female. Among SMI-positive patients, 108/186 (58%) were male, compared to 78/186 (42%) female. In the SMI-negative group, 94/202 (48%) were male, while 108/202 (52%) were female. The p-value for gender (0.222) indicates no significant difference in the prevalence of SMI between males and females. This suggests that gender does not substantially influence the likelihood of SMI in diabetic patients in this study. Nonetheless, the slight difference observed may warrant further investigation into gender-specific risk factors for SMI.

Table 2 illustrates the increasing prevalence of silent myocardial ischemia (SMI) among the 388 study participants over a 24-month follow-up period. At baseline, 186 participants (48%) were diagnosed with SMI, as identified by a 12-lead ECG. Over time, this prevalence rose significantly, reaching 210 participants (54%) at the 6-month mark (p=0.045), 220 participants (57%) at 12 months (p=0.032), 240 participants (62%) at 18 months (p=0.028), and 250 participants (64%) at 24 months (p=0.025). The diagnoses during follow-up were confirmed through various diagnostic tests, including treadmill stress tests and myocardial perfusion imaging (MPI).

**Table 2:** Changes in prevalence of silent myocardial ischemia over time Diagnosis of SMI was confirmed using 12-lead ECG at baseline and treadmill stress tests (TST) with myocardial perfusion imaging (MPI) during follow-ups (6, 12, 18, and 24 months). Prevalence percentages and confidence intervals (CIs) were calculated to ensure comparability: baseline (48%, 95% CI 43.3–52.6%), 6 months (54%, 49.5–58.7%), 12 months (57%, 52.3–61.6%), 18 months (62%, 57.5–66.5%), and 24 months (64%, 59.6–68.3%), all statistically significant (p<0.05). Statistical analysis adjusted for confounders like age, gender, diabetes duration, HbA1c, smoking status, and BMI. The increase in SMI prevalence likely reflects disease progression, cumulative risk exposure, and improved detection via advanced modalities, aligning with trends in high-risk diabetic populations.

Follow-Up Period	SMI Diagnosed (N)	Prevalence of SMI (%)	p-value
Baseline	186	48%	-
6 Months	210	54%	0.045
12 Months	220	57%	0.032
18 Months	240	62%	0.028
24 Months	250	64%	0.025

The p-values indicate statistically significant increases in SMI prevalence at each follow-up interval, underscoring the progressive nature of this condition in high-risk diabetic populations. This trend highlights the critical importance of regular monitoring for SMI in diabetic patients.

The observed 16% rise in SMI prevalence over the 24-month period (from 186 to 250 participants) emphasizes the need for early and continuous surveillance to detect SMI and implement timely interventions. The findings support the necessity for ongoing screening and preventive measures to mitigate the cardiovascular risks associated with diabetes and the progression of SMI.

Table 3 illustrates the prevalence of SMI among 388 diabetic participants. The overall prevalence of SMI in this cohort was found to be 186 (48%), indicating that nearly half of the diabetic population in the study exhibited signs of this condition. Among the 38 participants with microalbuminuria, the prevalence of SMI was higher (n=22; 58%), reflecting a significant correlation between microalbuminuria and SMI. In contrast, the prevalence of SMI in diabetic patients without microalbuminuria (350 participants) was lower (n=164; 36%). This significant difference suggests that microalbuminuria may serve as an important predictor of SMI, highlighting the need for targeted screening and monitoring in this high-risk group.

**Table 3:** Prevalence of silent myocardial ischemia in diabetic patients SMI: Silent myocardial ischemia n (%): Number of participants (percentage) Chi-square test: Used to calculate p-value and compare the prevalence of SMI between groups with and without microalbuminuria. Odds Ratio (OR): 2.43 (95% CI: 1.26–4.67), indicating increased SMI risk in participants with microalbuminuria. p-value: Indicates the statistical significance of differences between groups (<0.05 considered significant).

Characteristic		Value
Total Participants		388
Prevalence of SMI (n; %)		n=186/388; 48%
Prevalence of SMI in Diabetic Patients with Microalbuminuria (n; %)		n=22/38; 58%
Prevalence of SMI in Diabetic Patients without Microalbuminuria (n; %)		n=164/350; 36%
Gender	Male (n; %)	n=93/186; 50%
	Female (n; %)	n=93/186; 50%
p-value		<0.05

Interestingly, the gender distribution among those diagnosed with SMI was balanced, with the cases being male (n=93; 50%) and female (n=93; 50%). This suggests that the prevalence of SMI does not significantly differ by gender in this cohort. The statistical significance of the microalbuminuria findings (p-value < 0.05) emphasizes the importance of considering microalbuminuria as a potential predictor of silent myocardial ischemia in diabetic patients.

These data underline the critical need for routine screening and monitoring for SMI, particularly among individuals with microalbuminuria, to improve cardiovascular outcomes in this high-risk group. While gender did not appear to influence the prevalence of SMI in this study, further research exploring other gender-specific factors in larger, more diverse populations is warranted.

The multivariate logistic regression analysis, presented in Table 4, identifies key predictors associated with silent myocardial ischemia (SMI) in diabetic patients. The results demonstrate that the duration of diabetes significantly increased the odds of SMI, with an odds ratio (OR) of 1.28 (95% CI: 1.11 - 1.47, p=0.001). This indicates that for each additional year of diabetes, the risk of SMI increased by 28%.

**Table 4:** Multivariate logistic regression analysis of predictors for silent myocardial ischemia OR: Odds ratio; CI: Confidence interval; CAD: Coronary artery disease. All continuous variables (duration of diabetes, HbA1c, age, and BMI) were analyzed as continuous variables in the logistic regression model. The statistical significance of predictors was evaluated using Wald chi-square tests within the logistic regression framework. Model goodness-of-fit was assessed using the Hosmer-Lemeshow test (p=0.78), confirming adequate model fit. p-value less than 0.05 was significant.

Predictor	Odds Ratio (OR)	95% Confidence Interval (CI)	p-value
Duration of Diabetes (years)	1.28	1.11 – 1.47	0.001
HbA1c (%)	1.75	1.32 – 2.32	<0.001
Age (years)	1.05	1.01 – 1.10	0.035
Gender (Male vs. Female)	1.2	0.85 – 1.70	0.222
Smoking Status (Yes vs. No)	1.42	1.05 – 1.92	0.025
Microalbuminuria (Yes vs. No)	2.89	1.64 – 5.07	<0.001
Family History of CAD (Yes vs. No)	1.35	0.94 – 1.95	0.086
BMI (per kg/m²)	1.12	1.01 – 1.25	0.032

Hemoglobin A1c (HbA1c) levels also emerged as a significant predictor of SMI, with an OR of 1.75 (95% CI: 1.32 - 2.32, p<0.001). This suggests that higher HbA1c levels substantially elevate the risk of SMI, underscoring the crucial role of glycemic control in preventing cardiovascular complications in diabetic patients.

Age was another significant factor influencing the odds of SMI, with an OR of 1.05 (95% CI: 1.01 - 1.10, p=0.035), indicating a 5% increase in SMI risk for each year of age. Additionally, current smoking status was associated with a 42% increase in the odds of SMI (OR: 1.42, 95% CI: 1.05 - 1.92, p=0.025), highlighting smoking as a modifiable risk factor for SMI in this cohort.

Microalbuminuria showed the strongest association with SMI, with an OR of 2.89 (95% CI: 1.64 - 5.07, p<0.001). This suggests that diabetic patients with microalbuminuria are nearly three times more likely to experience SMI than those without microalbuminuria, emphasizing the importance of screening for microalbuminuria as part of routine care for diabetic patients.

While a family history of coronary artery disease (CAD) was associated with an OR of 1.35 (95% CI: 0.94 - 1.95, p=0.086), this association did not reach statistical significance. Interestingly, gender was not a significant predictor of SMI, with an OR of 1.20 (95% CI: 0.85 - 1.70, p=0.222), suggesting that gender differences may not substantially influence the risk of SMI in this study population.

Additionally, body mass index (BMI) showed a significant association with SMI, with an OR of 1.12 (95% CI: 1.01 - 1.25, p=0.032). This suggests that for each unit increase in BMI, the odds of SMI increase by 12%, indicating the relevance of maintaining a healthy weight to reduce the risk of SMI in diabetic patients.

These findings emphasize the importance of monitoring key clinical factors, such as the duration of diabetes, glycemic control (HbA1c), and the presence of microalbuminuria, in identifying diabetic patients at higher risk for silent myocardial ischemia. Targeted interventions and close follow-up are crucial for this high-risk group to reduce the likelihood of adverse cardiovascular events.

The odds ratios reported in Table 4 highlight the strong associations between several clinical factors and SMI. For instance, an increase of 1% in HbA1c levels is associated with a 75% increase in the odds of SMI, underscoring the significant impact of glycemic control on reducing the risk of silent myocardial ischemia in diabetic patients.

The association between microalbuminuria (OR = 2.89) and SMI is especially compelling, suggesting that diabetic patients with microalbuminuria are nearly three times more likely to experience SMI compared to those without microalbuminuria. This reinforces the need for routine screening of microalbuminuria in diabetic patients as part of a comprehensive approach to prevent SMI and other cardiovascular complications.

**Figure 1:**
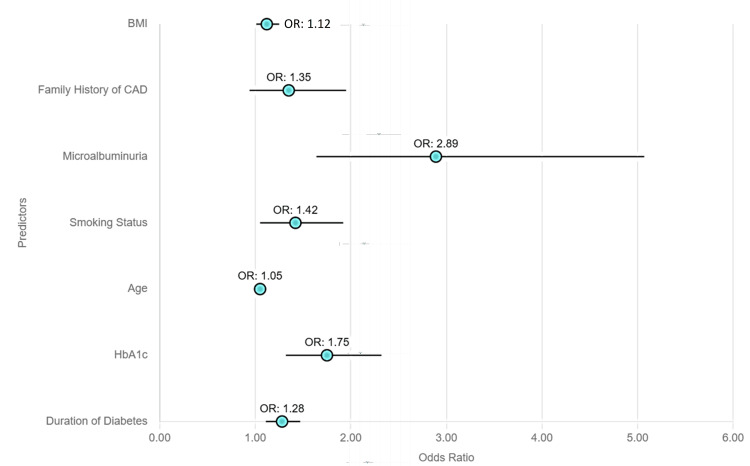
Scatter plot showing the predictors of silent myocardial ischemia

Figure 1 highlights that the prevalence of silent myocardial ischemia (SMI) was notably higher in diabetic patients with microalbuminuria. Specifically, 22/38 (58%) of patients with microalbuminuria exhibited SMI, compared to 164/350 (36%) of those without microalbuminuria. This difference emphasizes the strong association between microalbuminuria and the increased likelihood of SMI.

Multivariate logistic regression analysis identified several key predictors of SMI in this population. The duration of diabetes was significantly associated with an increased risk of SMI, with an odds ratio (OR) of 1.28 (95% CI: 1.11 - 1.47, p=0.001), indicating a 28% increased risk of SMI for each additional year of diabetes. HbA1c levels were another critical predictor, with an OR of 1.75 (95% CI: 1.32 - 2.32, p<0.001), showing that higher HbA1c levels significantly elevate the risk of SMI. Age also played a role, with an OR of 1.05 (95% CI: 1.01 - 1.10, p=0.035), suggesting that for every year of increased age, the risk of SMI rose by 5%.

Furthermore, smoking status was associated with a 42% increased likelihood of SMI (OR 1.42, 95% CI: 1.05 - 1.92, p=0.025), underscoring the detrimental impact of smoking on heart health in diabetic individuals. Notably, the presence of microalbuminuria emerged as the strongest predictor of SMI, with an OR of 2.89 (95% CI: 1.64 - 5.07, p<0.001), indicating that diabetic patients with microalbuminuria were nearly three times more likely to develop SMI compared to those without.

Although the presence of a family history of coronary artery disease (CAD) showed a trend towards an increased risk of SMI (OR 1.35, 95% CI: 0.94 - 1.95, p=0.086), it did not reach statistical significance. This suggests that while a family history of CAD may contribute to the risk of SMI, further investigation is required to fully understand its role in SMI development.

These findings underscore the importance of incorporating risk factors such as microalbuminuria, diabetes duration, HbA1c levels, and smoking status into SMI screening strategies. Early identification of at-risk individuals through comprehensive monitoring can help facilitate timely interventions to reduce cardiovascular complications in the diabetic population.

## Discussion

The current findings corroborate previous studies, particularly the significant role of age, prolonged diabetes duration, and poor glycemic control in the development of SMI. In this study, older age and longer duration of diabetes were associated with an increased prevalence of SMI, which aligns with prior research showing that age and diabetes duration are crucial predictors of cardiovascular complications among diabetic patients [5,6]. Specifically, the mean age of 60.4 years in our study is consistent with studies suggesting that the risk of cardiovascular events, including SMI, increases significantly in older adults, especially those with chronic conditions like diabetes. The association between prolonged diabetes duration and SMI prevalence can be explained by the long-term effects of hyperglycemia, which lead to vascular changes such as atherosclerosis and endothelial dysfunction, predisposing individuals to ischemic events [12]. This is supported by studies demonstrating that prolonged exposure to elevated blood glucose can induce oxidative stress, inflammatory pathways, and vascular damage, which are central to the pathogenesis of cardiovascular diseases [13,14].

The significant relationship between higher HbA1c levels and SMI in our study further emphasizes the importance of glycemic control in preventing cardiovascular complications. Higher HbA1c levels (9.1% vs. 7.9% in SMI-positive vs. SMI-negative groups) reflect poor long-term glycemic control and are consistent with findings from other studies that show a direct correlation between elevated HbA1c and the risk of both microvascular and macrovascular complications in diabetic patients [15]. Poor glycemic control accelerates the development of atherosclerosis and impairs endothelial function, contributing to the increased risk of ischemic heart disease and SMI. These results underscore the need for intensive glycemic management to reduce cardiovascular risk among diabetic patients, which is supported by studies indicating that tighter glycemic control can reduce the incidence of cardiovascular events in diabetes [16,17].

Our findings also highlight the significant role of smoking in the development of SMI. The prevalence of smoking was higher in the SMI-positive group (30%) compared to the SMI-negative group (19%), which is consistent with the body of literature identifying smoking as a major risk factor for ischemic heart disease in diabetic patients [18]. Smoking accelerates atherosclerosis, exacerbates endothelial dysfunction, and increases inflammatory markers, all of which contribute to the development of SMI. Although smoking status was statistically significant in our study (p=0.034), the non-significant trend observed for physical activity (p=0.075) warrants further attention. While physical activity did not reach conventional levels of statistical significance, it is important to acknowledge that the p-value of 0.075 suggests a potential association that may become significant in larger studies or with more precise measures of physical activity. The trend toward lower physical activity in the SMI-positive group (35% vs. 45%) supports the growing body of evidence linking physical inactivity with cardiovascular risk in diabetes. In future studies, larger sample sizes and objective measures of physical activity may provide clearer insights into the role of physical activity in reducing SMI risk among diabetic patients.

Microalbuminuria was also identified as a significant predictor of SMI in our study, with patients who had microalbuminuria exhibiting a higher prevalence of SMI (58%) compared to those without (36%). This finding is consistent with existing literature that highlights microalbuminuria as a marker of endothelial dysfunction and increased cardiovascular risk in diabetic patients [19]. The significant odds ratio (OR = 2.89, p<0.001) for microalbuminuria underscores the importance of early detection and management of this condition, as it serves as a powerful indicator of cardiovascular risk. Other studies have shown that microalbuminuria reflects systemic vascular damage and is strongly associated with an increased risk of cardiovascular events, making it a critical target for early intervention [20]. The use of medications like ACE inhibitors or angiotensin receptor blockers (ARBs) to manage microalbuminuria could help reduce both renal and cardiovascular risks, aligning with evidence that controlling renal dysfunction improves cardiovascular outcomes in diabetes [16,21].

Although a family history of CAD did not achieve statistical significance in our study (OR = 1.35, p=0.086), it is still an important factor to consider. Family history is often a strong predictor of cardiovascular disease, and its lack of significance in this study may be attributed to sample size limitations or potential interaction effects with other variables such as glycemic control, smoking, and diabetes duration. Several studies have shown that a family history of CAD is a significant predictor of ischemic heart disease, suggesting that larger or more stratified studies could further elucidate its role in predicting SMI in diabetic populations [22,23]. Similarly, the lack of significance for gender (p=0.222) is noteworthy. While gender differences in cardiovascular risk factors are well-documented, the absence of a significant gender effect in this study could indicate that the risk factors for SMI in diabetes are more pronounced in both genders equally. However, gender-specific studies may reveal nuanced differences that warrant further investigation, especially in relation to hormonal influences on cardiovascular health in diabetic individuals [24].

Our study provides several actionable recommendations based on the findings. First, stringent glycemic control should be maintained, with HbA1c targets ideally below 7%. However, individualized goals may be necessary for high-risk patients to balance the benefits of reducing cardiovascular events with the potential risks of hypoglycemia. Effective glycemic management remains a cornerstone in mitigating cardiovascular complications among diabetic patients.

Second, smoking cessation should be prioritized as a key component of diabetes care. Tailored smoking cessation interventions, such as pharmacotherapy and behavioral counseling, should be widely implemented. Given the strong association between smoking and cardiovascular risk, targeted strategies to support quitting are essential to improving outcomes in this population.

Third, promoting physical activity is highly recommended. Although our study did not find a statistically significant association between physical activity and SMI risk, the observed trend suggests that further investigation is warranted. Regular exercise remains an important preventive strategy for reducing cardiovascular risk and improving overall health in diabetic patients.

Finally, routine screening for microalbuminuria should be integrated into standard diabetes care protocols. Early identification of microalbuminuria allows for timely interventions that can prevent the progression of cardiovascular and renal complications. Given its role as a marker of endothelial dysfunction and cardiovascular risk, microalbuminuria screening represents a valuable tool in comprehensive diabetes management.

From a public health perspective, these findings highlight the importance of comprehensive cardiovascular screening for diabetic patients, especially those with risk factors such as poor glycemic control, smoking, and microalbuminuria. Public health initiatives should aim to improve access to diabetes care while promoting lifestyle modifications, including smoking cessation and increased physical activity. Policy changes could be instrumental in ensuring routine screening for microalbuminuria and implementing targeted interventions to address cardiovascular risk in this population.

Strengths, limitations, and future perspectives

This study has notable strengths, including a comprehensive assessment of demographic, clinical, and biochemical factors associated with SMI in a well-defined diabetic population. The use of objective diagnostic tools, such as ECG and stress testing, enhances the accuracy of SMI detection and strengthens the study's validity. These tools are well-established in clinical practice, providing reliable and clinically relevant insights.

However, several limitations should be considered. The observational design inherently limits causal inferences, and reliance on self-reported data for variables like smoking status and physical activity may introduce recall bias. Additionally, the relatively modest sample size may have restricted the statistical power to detect smaller associations, such as the impact of family history of CAD and physical activity trends. The cross-sectional nature of the study prevents establishing temporal relationships between risk factors and SMI, while the single-center setting may limit generalizability to broader populations.

To address these limitations, several steps are recommended. Future research should employ longitudinal designs to explore causal relationships and disease progression more effectively. Expanding the sample size and recruiting participants from diverse geographic and demographic backgrounds would enhance representativeness and external validity. Utilizing validated tools, such as wearable devices, to objectively measure lifestyle factors like physical activity can reduce reliance on self-reported data and minimize bias. Finally, incorporating advanced statistical methods to account for potential confounders and interaction effects, as well as exploring novel biomarkers for SMI, would provide more robust and actionable insights.

Future research should focus on expanding multicenter studies to further elucidate the causal relationships between identified risk factors and the progression of SMI in diabetic patients. Investigating the role of age-specific differences in SMI risk and analyzing the influence of diabetes medications could provide a more nuanced understanding of disease progression and intervention strategies. Moreover, exploring potential interventions, such as tailored glycemic control protocols, pharmacological therapies, and targeted lifestyle modifications, aimed at reducing cardiovascular risk in this high-risk population is essential. Large-scale, geographically diverse studies are warranted to address the limitations of generalizability and provide robust evidence for policy updates and clinical guidelines.

## Conclusions

The findings of this study reveal that nearly 50% of participants with diabetes exhibited SMI, with a notably higher prevalence in those with microalbuminuria. Key predictors of SMI identified through multivariate analysis include prolonged diabetes duration, elevated HbA1c levels, increased age, current smoking status, and the presence of microalbuminuria. These factors significantly increase the risk of cardiovascular complications, with microalbuminuria demonstrating a nearly threefold increase in SMI risk. The high prevalence of SMI in this cohort has important implications for the broader diabetic population and healthcare systems, highlighting the urgent need for routine screening and early intervention. Early identification of individuals at risk, particularly those with additional risk factors such as prolonged diabetes and poor glycemic control, is essential for preventing the progression of cardiovascular diseases. Targeted interventions, including intensive glycemic control protocols, smoking cessation programs, and regular monitoring of microalbuminuria, should be integral components of diabetes care.

Moreover, the findings underscore the need for updated clinical guidelines and policy changes that advocate for more systematic cardiovascular risk assessments in diabetic populations. These efforts could contribute to more personalized care and help mitigate the cardiovascular burden in this high-risk group. This study calls for further research, particularly longitudinal studies, to explore the causal relationships between the identified risk factors and disease progression. Additionally, multicenter studies are needed to confirm these findings across diverse populations and help refine strategies for cardiovascular risk reduction in diabetic patients.
